# Overexpression of Human Estrogen Biosynthetic Enzyme Hydroxysteroid (17beta) Dehydrogenase Type 1 Induces Adenomyosis-like Phenotype in Transgenic Mice

**DOI:** 10.3390/ijms23094815

**Published:** 2022-04-27

**Authors:** Taija Heinosalo, Kalle T. Rytkönen, Niina Saarinen, Päivi Järvensivu, Pauliina Damdimopoulou, Leena Strauss, Satu Orasniemi, Petricia Horshauge, Michael Gabriel, Pasi Koskimies, Claes Ohlsson, Pauliina Kronqvist, Matti Poutanen

**Affiliations:** 1 Research Centre for Integrative Physiology and Pharmacology, Institute of Biomedicine, University of Turku, Kiinamyllynkatu 10, 20520 Turku, Finland; katury@utu.fi (K.T.R.); niina.saarinen-aaltonen@forendo.com (N.S.); paijarv@gmail.com (P.J.); leesal@utu.fi (L.S.); satu.orasniemi@gmail.com (S.O.); petriciahorshauge@gmail.com (P.H.); micawo@utu.fi (M.G.); 2 Turku Bioscience Centre, University of Turku and Åbo Akademi University, Tykistökatu 6, 20520 Turku, Finland; 3 Forendo Pharma, Itäinen Pitkäkatu 4, 20520 Turku, Finland; pasi.koskimies@forendo.com; 4 Division of Obstetrics and Gynecology, Department of Clinical Science, Intervention and Technology, Karolinska Institutet and Karolinska University Hospital Huddinge, 141 86 Stockholm, Sweden; pauliina.damdimopoulou@ki.se; 5 Sahlgrenska Osteoporosis Centre, Centre for Bone and Arthritis Research, Department of Internal Medicine and Clinical Nutrition, Institute of Medicine, Sahlgrenska Academy, University of Gothenburg, 413 45 Gothenburg, Sweden; claes.ohlsson@medic.gu.se; 6 Department of Drug Treatment, Sahlgrenska University Hospital, Region Västra Götaland, 413 45 Gothenburg, Sweden; 7 Department of Pathology, University of Turku, Kiinamyllynkatu 10/MedD5A, 20500 Turku, Finland; paukro@utu.fi; 8 Turku Center for Disease Modeling (TCDM), University of Turku, 20014 Turku, Finland

**Keywords:** adenomyosis, HSD17B1, estrogen

## Abstract

Hydroxysteroid (17beta) dehydrogenase type 1 (HSD17B1) is an enzyme that converts estrone to estradiol, while adenomyosis is an estrogen-dependent disease with poorly understood pathophysiology. In the present study, we show that mice universally over-expressing human estrogen biosynthetic enzyme *HSD17B1* (HSD17B1TG mice) present with adenomyosis phenotype, characterized by histological and molecular evaluation. The first adenomyotic changes with endometrial glands partially or fully infiltrated into the myometrium appeared at the age of 5.5 months in HSD17B1TG females and became more prominent with increasing age. Preceding the phenotype, increased myometrial smooth muscle actin positivity and increased amount of glandular myofibroblast cells were observed in HSD17B1TG uteri. This was accompanied by transcriptomic upregulation of inflammatory and estrogen signaling pathways. Further, the genes upregulated in the HSD17B1TG uterus were enriched with genes previously observed to be induced in the human adenomyotic uterus, including several genes of the NFKB pathway. A 6-week-long HSD17B1 inhibitor treatment reduced the occurrence of the adenomyotic changes by 5-fold, whereas no effect was observed in the vehicle-treated HSD17B1TG mice, suggesting that estrogen is the main upstream regulator of adenomyosis-induced uterine signaling pathways. HSD17B1 is considered as a promising drug target to inhibit estrogen-dependent growth of endometrial disorders. The present data indicate that HSD17B1 over-expression in TG mice results in adenomyotic changes reversed by HSD17B1 inhibitor treatment and HSD17B1 is, thus, a potential novel drug target for adenomyosis.

## 1. Introduction

Adenomyosis is a common gynecological disease, where endometrial tissue is located inside the myometrium, the uterine layer surrounding the endometrium. Adenomyosis was previously primarily found in hysterectomy specimens from perimenopausal women, but due to improved imaging methods, it has become apparent that adenomyosis is also considerably present in women of reproductive age [1,2]. Based on imaging studies, adenomyosis is prevalent in ~20% of women searching gynecological aid [3]. Patients with adenomyosis experience abnormal uterine bleeding, pain and infertility [2,4]. Similar to endometriosis, there is no targeted medical treatment for adenomyosis, and it is typically treated with hormonal contraceptives generating endometrial atrophy [5]. Hysterectomy is also a treatment option in older women [1]. The etiopathogenesis of adenomyosis is poorly understood, but there is evidence supporting different theories regarding the origin of the disease. These include the theory of direct invasion of endometrium into the myometrium, the theory of metaplasia of stem cells in the myometrium and the theory of infiltration of retrograde menstrual effluent from the serosal side [2,4].

The involvement of various molecular mechanisms has been shown in the development of adenomyosis, including altered sex steroid signaling [4,6]. Adenomyosis typically occurs in the presence of estrogenic stimulation during the reproductive years, and is often associated with other estrogen-dependent disorders, such as endometriosis, leiomyoma and endometrial hyperplasia and cancer [2,4,7,8]. The use of selective estrogen receptor modulator (SERM), such as tamoxifen, is found to be a risk factor for adenomyosis in humans, and tamoxifen exposure also induces adenomyosis in mice [9,10]. Furthermore, upregulation of aromatase enzyme (cytochrome P450 family 19 subfamily A member 1; CYP19A1) mRNA and protein, which converts androgens to estrogens, has been reported in adenomyosis [11,12]. The dependence of adenomyosis phenotype on estrogens has also been demonstrated in a mouse model expressing dominantly stabilized beta-catenin (Ctnnb1) in the uterus [13].

In addition to the endocrine-mediated effects of steroid hormones produced de novo in the ovaries and adrenals, several peripheral tissues express steroidogenic enzymes, enabling local regulation of steroid hormone concentrations in an intracrine manner in these tissues [14,15]. Hydroxysteroid (17beta) dehydrogenase type 1 (HSD17B1) is one such enzyme, catalyzing the last step of estrogen biosynthesis converting the weak estrogen estrone (E1) to highly active estradiol (E2), both in steroid biosynthetic and peripheral tissues [15]. Different preclinical studies have shown that HSD17B1 is involved in local estrogen production in the endometrium and endometrial diseases, including endometriosis [15,16,17,18]. In the present study we investigated the role of HSD17B1 in adenomyosis by studying the uterine phenotype of transgenic mice over-expressing human HSD17B1 (HSD17B1TG mice) that also present with other estrogen-dependent phenotypic changes [16,19,20].

## 2. Results

### 2.1. Phenotypic and Transcriptomic Changes in the Uterus of HSD17B1TG Mice at the Age of 4 Months Indicate Enhanced Estrogen Action and Inflammation

**Figure 1:**
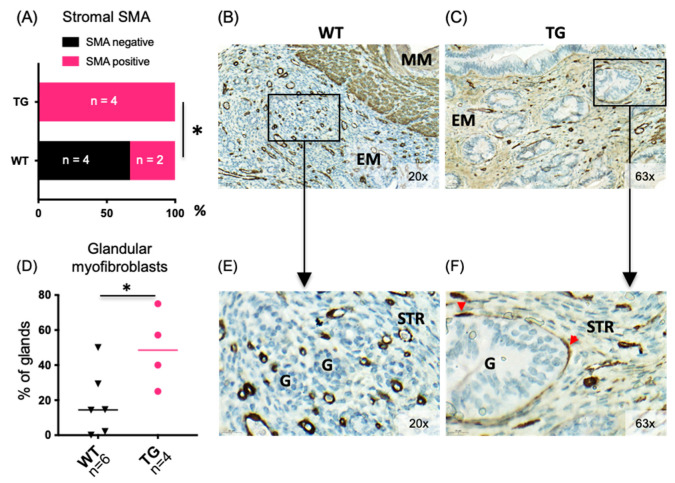
Uterine changes preceding the adenomyosis phenotype in HSD17B1TG females. At the age of four months, HSD17B1TG uterus showed changes related to mechanical stress and increased exposure to estrogens. (**A**) The endometrial stromal cells appeared more positive for smooth muscle actin (SMA), as shown by representative images of immunohistochemical SMA analysis for WT (**B**) and HSD17B1TG (**C**) uteri. Higher magnifications (of the areas indicated by black squares in (**B**,**C**) are shown in (**E**,**F**), respectively. (**D**) The endometrial glands in HSD17B1TG mice were surrounded by myofibroblast cells significantly more often than in WT mice. The SMA-positive myofibroblasts are shown by the red arrowheads in (**F**). * *p* < 0.05, MM = myometrium, EM = endometrium, G = gland, STR = stroma, triangle = WT, circle = TG.

Uterine samples of WT and HSD17B1TG mice were first studied at the age of 4 months and various changes suggestive of mechanical stress and estrogen exposure were observed between HSD17B1TG and WT mice. As shown in Figure 1A–C, an immunohistochemistry (IHC) analysis indicated that in the HSD17B1TG females the endometrial stroma overall appeared lightly positive for smooth muscle actin (SMA), in contrast to the mainly SMA-negative stroma in WT mice (*p* = 0.035).

The endometrial stroma was SMA-positive in 100% HSD17B1TG mice when analyzed at the age of 4 months, while SMA-positivity was only observed in one third of WT mice. Furthermore, a significant proportion of the endometrial glands in HSD17B1TG females were surrounded by an SMA-positive myofibroblast cells, whereas these cells did not surround the glands in WT mice to the same extent, as visually quantitated from the scanned IHC sections. 18 ± 8% of glands per mouse in WT and 49 ± 11% of glands in HSD17B1TG females were surrounded by the myofibroblast cells (*p* = 0.042; Figure 1D,F).

**Figure 2:**
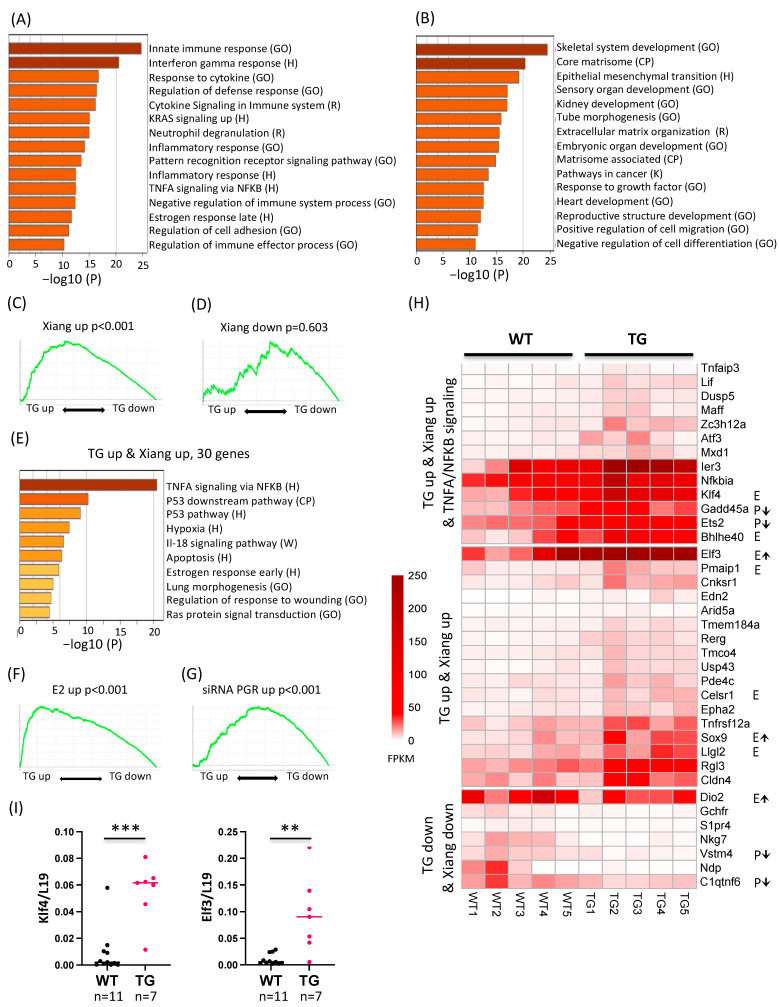
Transcriptomic uterine changes in HSD17B1TG females at the age of 4 months preceding adenomyosis development. Functional enrichment analysis of genes that were upregulated (**A**) or downregulated (**B**) in HSD17B1TG (*n* = 5) versus WT (*n* = 5) uteri. Differentially regulated genes (FDR < 0.05, FC > 2, > 1 FPKM) were used as an input in metascape.org with functional categories from GO Biological Processes (GO), Hallmark gene sets (H), canonical pathways (CP), KEGG (K), Reactome (R) and WikiPathways (W). (**C**) Gene Set Enrichment Analysis (GSEA) of transcriptionally upregulated genes previously detected in the eutopic endometrium of human adenomyosis patients compared to controls [21] (FDR < 0.05, FC > 2) showed a significant overlap with the upregulated genes in HSD17B1TG uterus compared with WT found in this study, whereas genes downregulated in human adenomyotic eutopic endometrium were not enriched in the downregulated genes in the HSD17B1TG mice (**D**). (**E**) Functional enrichment analysis of the 30 genes upregulated (FDR < 0.05, FC > 2) in both HSD17B1TG uterus and human eutopic endometrium from adenomyosis patients [21]. (**F**) GSEA of transcriptionally upregulated genes in estradiol (E2)-treated mouse uterus [22] (FDR < 0.05, FC > 2) on the ranked gene changes in HSD17B1TG uteri identified in the present study (positive fold change on the left), supporting enhanced estrogen action in the HSD17B1TG uterus. (**G**) GSEA using genes upregulated by siRNA targeting progesterone receptor (siPGR; FDR < 0.01) [23] on the ranked gene changes in HSD17B1TG females observed in this study (positive fold change on the left). The data show a significant overlap for genes upregulated in HSD17B1TG uterus and by the siPGR. (**H**) A heatmap showing transcriptomic changes between HSD17B1TG and WT uteri (FDR < 0.05, FC < 2) for genes, which also have concordant transcriptomic change in the eutopic endometrium from human adenomyosis patients [21]. In the top panel, genes of the TNFα signaling via NFKB are shown, being the top enriched Hallmark category. Estrogen regulation of the genes is indicated with E and an arrow for the genes shared with Hallmark estrogen response early/late term (M5906, M5907) and for the genes from Damdimopoulou et al., 2011 [22]. Those genes shown to be repressed by progesterone (siPGR-upregulated) [23] are marked with P. Transcription is displayed using fragments per kilobase of transcript sequence per millions base pairs sequenced (FPKM). (**I**) For *Klf4* and *Elf3,* transcriptomic analysis was confirmed by a larger number of individuals with RT-qPCR (WT = 11, TG = 7, ** *p* < 0.01, *** *p* < 0.001). Enrichment analysis output gene lists are shown in Supplementary Table S1C–E, GSEA input gene lists in Table S1F and GSEA output parameters in Table S1G.

With RNA sequencing, we detected 906 upregulated and 666 downregulated genes (FDR < 0.05, FC > 2, > 1 FPKM) in the pre-adenomyotic HSD17B1TG uterus compared to WT uterus at the age of 4 months (Supplementary Table S1A,B). Functional enrichment analysis of the upregulated genes indicated categories such as innate “immune response” and “other inflammation related pathways”, as well as “regulation of adhesion” and “late estrogen response” (Figure 2A). HSD17B1TG females do not cycle and are in the phase of constant estrogen stimulation [16], being in line with the late estrogen response. For the downregulated genes, functional categories such as “core matrisome” and “epithelial mesenchymal transition” were detected (Figure 2B).

We next compared the transcriptional changes in the HSD17B1TG uterus to those describing changes in eutopic endometrium in human adenomyosis patients [21], in mice treated with E2 [22] and in stromal cell cultures treated with siRNA against progestin receptor (PGR) [23], using gene set enrichment analysis (GSEA). GSEA indicated that genes upregulated in human eutopic endometrium of adenomyosis patients vs. normal endometrium [21] were enriched among HSD17B1TG-upregulated genes, whereas genes downregulated in human adenomyotic endometrium were not enriched (Figure 2C,D). The functional enrichment analysis of the 30 genes upregulated (FDR < 0.05, FC > 2) in both these datasets indicated functional categories including “TNFα signaling via NFKB”, “hypoxia”, “apoptosis”, “late estrogen response” and “wounding responses” (Figure 2E), suggesting that these endometrial pathways may be central in promoting adenomyosis development. More than third of the of the genes in this intersect (13/30) were part of TNFα signaling via NFKB term.

GSEA of genes previously shown to be upregulated by E2 in the mouse uterus [22] were also enriched among HSD17B1TG upregulated genes (Figure 2F), indicating that E2 response is hyperactive in the HSD17B1TG uterus. Furthermore, GSEA of genes upregulated in siRNA knockdown of PGR [23] indicated that these were enriched among HSD17B1TG-upregulated genes (Figure 2G). This suggests that progesterone-dependent gene repression is defective in the HSD17B1TG uterus, supported by our previous findings of anovulatory ovaries lacking progesterone-secreting corpora lutea in HSD17B1TG mice [16]. As shown in the heatmap (Figure 2H), the estrogen-regulated genes *Klf4*, *Bhlhe40*, *Elf3*, *Pmaip1*, *Celsr1*, *Sox9*, *Llgl2* and *Dio2* were upregulated in HSD17B1TG vs. WT uterus and in the dataset from Xiang et al., 2019 [21], suggesting that these may be central genes in the estrogen-dependent adenomyosis development both in human and mouse. Similarly, the PGR-repressed genes *Gadd45a*, *Ets2*, *Vstm4* and *C1qtnf6* may be important genes related to progesterone resistance-mediated mechanisms of adenomyosis development. Genes in the TNFα signaling via NFKB included both estrogen-upregulated (*Klf4*, *Bhlhe40)* and PGR downregulated (*Gadd45a*, *Ets2*) genes suggesting that the NFKB-mediated inflammatory responses in HSD17B1TG mouse uterus and human adenomyotic uterus may be primed by hormonal regulation.

We selected two of the above-mentioned genes with high transcription levels (*Klf4* and *Elf3)* for transcriptomic validation with RT-qPCR (Figure 2I), and the data confirmed *Klf4* and *Elf3* being significantly upregulated in HSD17B1TG compared to the WT uterus. The fold changes between the means in HSD17B1TG and WT mice were six times and nine times higher for *Klf4* (*p* < 0.001) and *Elf3* (*p* < 0.005), respectively.

### 2.2. HSD17B1TG Mice Develop an Adenomyosis-like Phenotype at the Age of 5.5 Months

**Figure 3:**
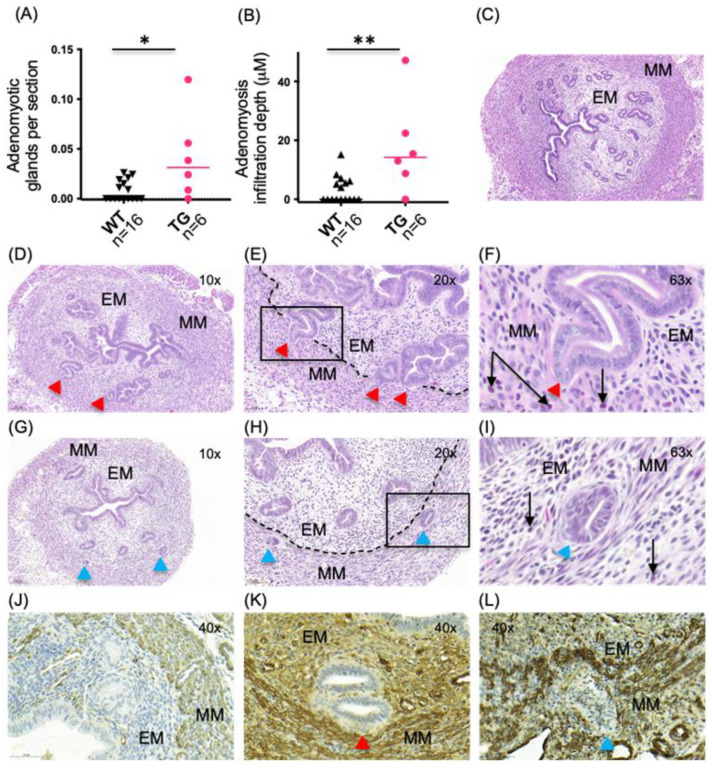
Adenomyotic changes are increased in HSD17B1TG females at the age of 5.5 months. (**A**) Significantly increased numbers of adenomyotic glands per analyzed sections were observed in HSD17B1TG mice compared to WT mice. (**B**) The infiltration depth was also significantly higher in HSD17B1TG mice compared to WT mice. (**C**) A cross-section of WT uterus without adenomyotic changes. (**D**) A cross-section of HSD17B1TG uterus with infiltrating glands. (**E**) A higher magnification of (**D**) with the endometrial and myometrial border highlighted with dashed line. (**F**) A higher magnification of (**E**), showing an infiltrating gland and eosinophils. (**G**) Few fully infiltrated glands were also observed in the HSD17B1TG mice. (**H**) A higher magnification of the section shown in Figure (**G**), with the endometrial–myometrial border highlighted with dashed line. (**I**) Eosinophils in the HSD17B1TG uterus. (**J**) Endometrial stroma of the WT uterus was negative in immunohistochemistry for smooth muscle actin (SMA). (**K**) SMA staining shows a partially infiltrated gland in HSD17B1TG mice. (**L**) SMA-staining showing fully infiltrated HSD17B1TG gland. The SMA-positive endometrial stroma is apparent in (**K**,**L**). * *p* < 0.05, ** *p* < 0.01, EM = endometrium, MM = myometrium, red arrowheads = partially infiltrated glands, blue arrowheads = fully infiltrated glands, black arrows = eosinophils, black squares = the areas shown in higher magnification, triangle = WT, circle = TG.

At the age of 5.5 months, adenomyotic changes were observed in HSD17B1TG females, as shown in Figure 3, whereas these changes were absent at the age of 4 months. Additionally, the WT females developed minor adenomyotic changes by the age of 5.5 months, but the observed changes were smaller and occurred at low frequency. The prevalence of the adenomyosis phenotype in the HSD17B1TG females was 83% (*n* = 5/6), while it was 44% (*n* = 7/16) in the WT females.

In HSD17B1TG females, on average, 8.2 ± 3.54 adenomyotic glands were observed per 1 mm uterine length, whereas only 1.8 ± 0.69 glands were present in the corresponding WT mice per 1 mm uterus (*p* = 0.028). Similarly, when the adenomyotic gland number was normalized by the number of histological sections analyzed, 4 ± 1.8 adenomyotic glands per 100 sections analyzed were found in the HSD17B1TG females and 0.8 ± 0.22 adenomyotic glands per 100 sections were found in the WT females (*p* = 0.035; Figure 3A).

**Figure 4:**
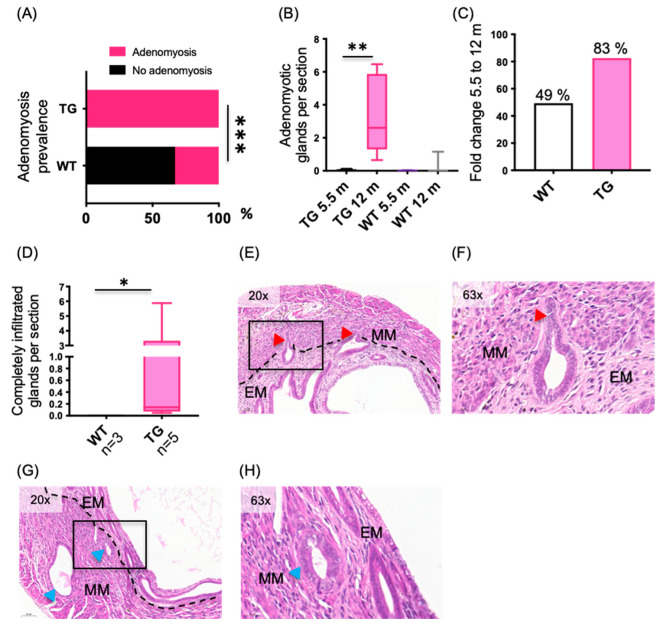
Increased amount of adenomyotic changes in HSD17B1TG mice at the age of 12 months. (**A**) When 43–57 serial sections per mice were analyzed, 100% of the HSD17B1TG females presented with adenomyotic changes at the 12 months of age (*n* = 5), while the changes were observed in 33% of the WT mice (*n* = 3). (**B**) Significantly increased numbers of adenomyotic glands per analyzed sections were observed in 12-month-old HSD17B1TG mice compared to the 5.5-month-old mice, whereas in WT mice, there was no difference between the two age groups. (**C**) The difference in the amount of adenomyotic glands between the HSD17B1TG and WT mice increased from 5.2-fold at the 5.5 months to 8.8-fold at the at the age of 12 months. (**D**) The number of adenomyotic glands completely infiltrated into the myometrium was significantly increased in HSD17B1TG mice compared to WT mice at the age of 12 months. (**E**) A cross-section of HSD17B1TG uterus showing partially infiltrated glands with invasive epithelial structures. (**F**) A higher magnification of (**E**). (**G**) A cross-section of HSD17B1TG uterus showing completely infiltrated glands. (**H**) A higher magnification of (**G**). * *p* < 0.05, ** *p* < 0.01, *** *p* < 0.001, EM = endometrium, MM = myometrium, red arrowheads = partially infiltrated glands, blue arrowheads = completely infiltrated glands, black squares = the areas shown in higher magnification.

The adenomyotic glands in the HSD17BTG females also showed significantly deeper myometrial infiltration depth. In the HSD17BTG females, the mean depth was 17.8 μm ± 6.06 vs. 3.2 μm ± 1.11 in WT females (*p* = 0.028, Figure 3B). Most of the adenomyotic changes observed at the age of 5.5 months showed partial infiltration (Figure 3D,E,K), but some completely infiltrated glands were also observed (Figure 3G–I,L). The stroma remained SMA-positive at the age of 5.5 months (Figure 3J–L). Different types of glandular infiltrations were observed: some glands retained their round shape when in contact with the myometrium (Figure 3I), whereas some glands showed invasive-type structures with sharp, collectively invasive epithelial ends reaching the myometrium (Figure 3F and Figure 4F). Immune cells were often observed at the site of infiltration (Figure 3F,I).

### 2.3. The Severity of the Adenomyosis Phenotype in the HSD17B1TG Is Advanced at Older Age

Compared to the age of 5.5 months, by the age of 12 months, the adenomyosis phenotype became more severe in the HSD17B1TG mice. As shown in Figure 4A, adenomyosis was prevalent in 100% of the HSD17B1TG mice (*n* = 5/5), while the incidence of adenomyotic changes in the WT mice remained at 33% (*n* = 1/3; *p* < 0.0001). The number of adenomyotic glands also increased significantly in HSD17B1TG mice over time, being 3.4 ± 1.19 glands per section at the age of 12 months (*p* = 0.007; Figure 4B), whereas in WT mice there was no increase in the number of adenomyotic changes per section over time. Thus, the difference in the adenomyotic gland between the WT and TG mice increased from 5.2- to 8.8-fold during aging (Figure 4C). There was also a tendency for an increased number of endometrial glands per section in the HSD17B1TG mice at the age of 12 months compared to WT females, but the difference was not statically significant. While glands completely infiltrated into the myometrium were only occasionally observed at the age of 5.5 months, their number was significantly higher in HSD17B1TG at the age of 12 months (1.4 ± 1.13 glands per section), while such infiltration was not observed in WT mice (*p* = 0.036, Figure 4D,G,H). In addition, partially infiltrated glands were observed (Figure 4E,F). Again, some infiltrated glands retained their round shape (Figure 4G,H), while some showed invasive epithelial structures (Figure 4E,F).

### 2.4. Circulating Levels of Estradiol Was Not Altered in the HSD17B1TG Females

**Figure 5:**
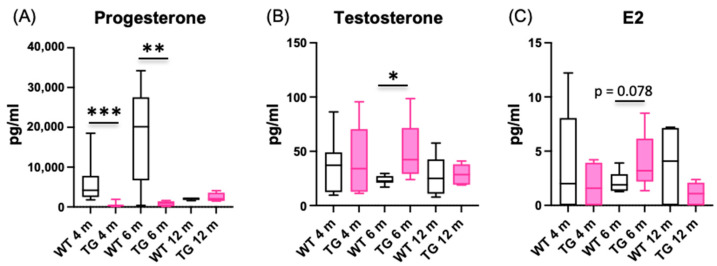
Serum hormone concentrations. Serum hormone concentrations were measured at the ages of 4, 6 and 12 months. (**A**) Serum progesterone level was significantly reduced at the ages of 4 and 6 months in HSD17B1TG females. (**B**) The concentration of serum testosterone was significantly increased in HSD17B1TG females at the age of 6 months. (**C**) There was no difference in serum E2 concentration in any time point. E2 = estradiol; 4 m WT *n* = 11, 4 m HSD17B1TG *n* = 7, 6 m WT *n* = 9, 6 m HSD17B1TG *n* = 5, 12 m WT *n* = 5, 12 m HSD17B1TG *n* = 7; * *p* < 0.05, ** *p* < 0.01, *** *p* < 0.001.

Serum hormone levels were measured at the ages of 4, 6 and 12 months in the female HSD17B1TG and WT mice. As shown in Figure 5, serum progesterone concentration was reduced at the ages of 4 months (WT 6043 ± 1729 pg/mL, HSD17B1TG 525 ± 239 pg/mL, *p* < 0.001) and 6 months (WT 18,057 ± 3883 pg/mL, HSD17B1TG 813 ± 293 pg/mL, *p* < 0.007). At the age of 6 months, significantly increased serum concentrations of testosterone (WT 23.5 ± 1.35 pg/mL, HSD17B1TG 48.7 ± 12.95 pg/mL, *p* = 0.021) were observed. There were no statistically significant differences in serum E2 concentration in any time point studied.

### 2.5. HSD17B1 Inhibition Rescues the Adenomyosis-like Phenotype in HSD17B1TG Mice

**Figure 6:**
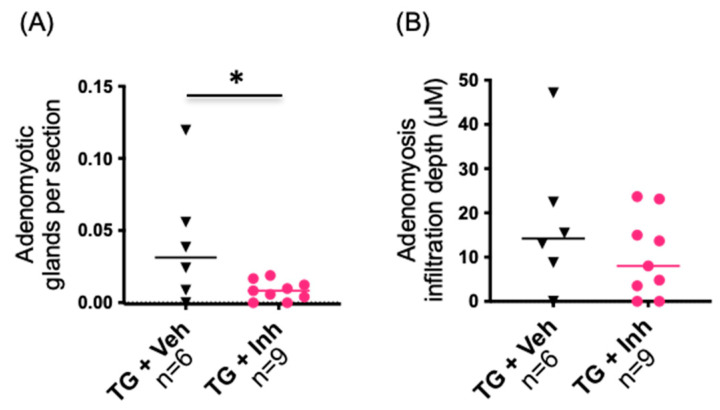
Prevention of the adenomyosis phenotype by an HSD17B1 inhibitor treatment. HSD17B1TG mice were treated with an HSD17B1 inhibitor for 6 weeks starting at the age of 4 months. (**A**) After the treatment, a significantly reduced number of adenomyotic glands per section were observed in HSD17B1TG mice compared to vehicle-treated mice. (**B**) The inhibitor treatment did not affect the infiltration depth of the glands. Veh = vehicle, Inh = HSD17B1 inhibitor, * *p* < 0.05, triangle = TG + Veh, circle = TG + Inh.

Although there was no statistically significant difference between the genotypes in circulating E2 concentration in any time point studied (Figure 5C), we tested the HSD17B1 dependence of the adenomyosis phenotype by treating the mice with an HSD17B1 inhibitor beta thiazole (compound no. 21 in Table 1 in Messinger et al., 2008 [24]). The mice were given 22 mg/kg/day HSD17B1 inhibitor for 6 weeks starting at the age of 4 months. As shown in Figure 6A, the treatment with the inhibitor reduced the number of adenomyotic glands 5-fold from that observed in the vehicle-treated HSD17B1TG mice (*p* = 0.042). However, the gland infiltration depth was not significantly altered by the HSD17B1 inhibitor (Figure 6B).

## 3. Discussion

Adenomyosis is a disease with poorly understood etiology, but several lines of evidence support estrogen-dependency of the disease [4,6]. HSD17B1 efficiently converts the weak estrogen E1 to the highly active E2, and is also able to convert androstenedione to testosterone. We have shown that overexpression of HSD17B1 in transgenic mice causes classical estrogen-dependent phenotypic changes, such as increased endometrial proliferation, endometrial hyperplasia and inflammation-aided rupture of the mammary gland myoepithelium [16,19,20]. In the present study, we show that HSD17B1TG mice also develop adenomyosis. At the age of 5.5 months, HSD17B1 showed an increased number of endometrial glands partially infiltrated into the myometrium, whereas at the age of 12 months, completely infiltrated glands were also present. The histology of some infiltrating glands showed signs for active, collective epithelial cell invasion, as described for cancer cells by Friedl et al. [25]. Thus, our data support the theory of direct, estrogen-dependent invasion of the basal endometrium into the myometrium.

In line with our data, several studies in mice have demonstrated that estrogen exposure also causes adenomyosis in mice [26]. Both prenatal and postnatal dosing of ethinyl-E2 were shown to induce adenomyosis in adult mice [27]. Neonatal dosing of the SERM compounds, such as tamoxifen and toremifene, also caused adenomyosis in adult mice [10,28] and the development of Ctnnb1-induced adenomyosis in mice required the presence of estrogen [13]. Our analysis of the serum steroid concentrations showed that the progesterone level was severely reduced in HSD17B1TG mice during the reproductive age (time points 4 and 6 months). We have previously shown that HSD17B1TG mice are anovulatory [16], and thus, the reduced progesterone concentration is explained by the lack of progesterone-producing corpora lutea in HSD17B1TG ovaries. These data were supported by upregulation of PGR-repressed genes [23] in the HSD17B1TG vs. WT uterus, as indicated by GSEA analysis. This further increases the estrogen effect on the endometrium, as the estrogen signaling is not opposed by progesterone.

We have also demonstrated that the androstenedione to testosterone conversion is increased in HSD17B1TG mice [29] and increased serum testosterone concentration was also observed at the age of 6 months in the present study. Although the serum E2 concentration was not significantly increased in HSD17B1TG mice, our previous studies have shown increased conversion of E1 to E2 in several tissues of HSD17B1TG females, including the uterus [16,19,20], suggesting a locally increased estrogen action in these tissues, without a change in the serum concentrations. This was also supported by our RNA-seq data, showing significant overlap in the previously reported estrogen-dependent genes in the mouse uterus [22] and the genes upregulated in the HSD17B1TG uterus. The pathways enriched among the upregulated genes in the HSD17B1TG uterus included inflammation, late estrogen response, KRAS signaling and regulation of cell adhesion, further supporting the hypothesis that these pathways are involved in the development of adenomyosis. Furthermore, we show that, similar to the other estrogen-dependent phenotypic changes in HSD17B1TG mice [16,19,20], the adenomyosis phenotype was prevented by the treatment with HSD17B1 inhibitor, confirming the dependence of the phenotype on the action of HSD17B1 and suggesting that increased estrogen exposure is upstream of all the altered adenomyosis-associated pathways.

Human adenomyotic uteri are hyperperistaltic [1,4,30]. According to one of the theories of adenomyosis pathogenesis, the hyperperistalsis causes microtrauma in the endometrial–myometrial junctional zone (EMJZ) that facilitates the invasion of basal endometrium into the myometrium [1,4,31]. The basal endometrial stromal cells in human adenomyotic uteri have been shown to express more SMA and have an increased number of concentrically arranged myofibroblast cells surrounding the basal glands, resulting from shearing stress caused by hyperperistalsis [32]. Similar to the human disease, HSD17B1TG mice show SMA-positive endometrial stromal cells already at the age of 4 months and spindle-shaped, SMA-positive myofibroblast cells were detected around the eutopic endometrial glands. These changes immediately preceded the adenomyosis phenotype in HSD17B1TG mice and were associated with prolonged estrogenic stimulation as indicated by the uterine upregulation of the estrogen-dependent genes in the HSD17B1TG uterus, such as *Klf4* and *Elf3* [22,33,34], the locally increased intra-uterine E2 biosynthesis and the endometrial hyperplasia phenotype [16].

We also observed significant overlap of 30 genes that are upregulated both in the pre-adenomyotic HSD17B1TG uterus and in human eutopic endometrium from adenomyosis patients [21]. The pathways enriched in the overlapping genes included TNFα signaling via NFKB, estrogen response, RAS-signaling and response to wounding, all also involved in adenomyosis development [35,36]. Thus, our data support the tissue injury and repair (TIAR)-theory of adenomyosis pathogenesis. Estrogen action has been shown to be positively associated with myometrial peristaltic contractility [37], causing the EMJZ microtrauma predisposing to adenomyosis, and our data also support the role of estrogens in the process.

By comparing our HSD17B1TG mouse data with previous studies [21,22,23], we observed that several of the genes in TNFα signaling via the NFKB category with the altered expression in the HSD17B1TG mice are also regulated by estrogens or progestins, suggesting that adenomyosis-associated inflammation may be hormonally primed in both HSD17B1TG uterus and human adenomyosis. Furthermore, increased NFKB p65, p50 and p52 protein expression has been observed in the endometrium of adenomyosis patients [38,39] and increased NFKB binding activity has been detected in adenomyotic stromal cells [40]. Additionally, NFKB and estrogen signaling have been reported to have additive interactions in endometrial epithelial cells [41], whereas PGR and NFKB have been reported to exhibit mutual repression [42], and decreased PGR-B expression has been observed in adenomyotic endometrium [38]. Similarly, PGR is a major anti-inflammatory factor repressing NFKB activation in myometrial cells [43]. Together, these observations suggest that estrogen-driven repression of PGR signaling together with the induction of NFKB signaling are potential drivers for adenomyosis, similar to that suggested for endometriosis [44], and may represent the link in hormonal and inflammatory signaling in both adenomyosis and endometriosis.

HSD17B1 inhibition is an attractive approach to reduce estrogen concentration locally in peripheral tissues. In preclinical studies performed by us and others in vivo, HSD17B1 inhibition was shown to reduce the growth of breast cancer xenografts [45,46,47,48], the development of inflammation-aided mammary epithelial rupture [20], the growth of endometrial cancer xenografts on chicken chorioallantoic membrane [49], the development of endometrial hyperplasia phenotype [16], the pain-related behavior of marmoset monkeys with endometriosis [50], the uterus weight induction and proliferation in uterotropic assay [19], the ERELuc reporter construct activity [19,20] and the serum E1 to E2 conversion [51]. In the present study, we showed that the development of the adenomyosis phenotype in HSD17B1TG females was prevented by the HSD17B1 inhibitor treatment. Adenomyosis is now understood to be a disease of both young and older women [1,52]. The conservative hysterectomy treatment is not an option for the majority of women in reproductive age, and therefore, new, targeted medical treatments are needed [52]. Because adenomyosis is an estrogen-dependent disease, HSD17B1 inhibition may be an option to reduce the estrogen action in adenomyosis lesions.

## 4. Materials and Methods

### 4.1. Breeding and Maintenance of HSD17B1TG Mice

The generation and maintenance of HSD17B1TG mouse line has been previously described [29]. Animal experiments were approved by the respective authorities, and the institutional policies on animal experimentation fully met the requirements as defined in the NIH Guide on animal experimentation. Briefly, FVB/N mice were made to universally over-express human *HSD17B1* cDNA under CMV-enhanced chicken beta actin promoter. HSD17B1TG males were bred with WT females to maintain the heterozygous mouse line and female mice with transgene expression were used in the study. The mice were housed under standard conditions with a 12 h light/darkness cycle at 21 ± 1 °C and free access to soy-free RM3 chow (Special Diet Service, Whitham Essex, UK) and tap water. Before sample collection, mice were terminally anesthetized with an intraperitoneal injection of 400–800 μL 2.5% tribromoethanol (Avertin; Aldrich Chemical Co., Milwaukee, WI, USA.) [53], and blood was withdrawn from the heart, followed by euthanasia by cervical dislocation. Blood was allowed to stand overnight at +4 °C and was centrifuged at 3000 rpm using a table centrifuge to separate serum. Tissues were weighed, snap-frozen in liquid N_2_ and stored at −80 °C or processed for histological analysis as described below.

### 4.2. Histomorphometric Analysis of the Uterus

The uteri were fixed in 4% paraformaldehyde at room temperature for 15–20 h, dehydrated and paraffin-embedded. HSD17B1TG mice do not cycle and WT uterus samples were collected at random cycle phases. Serial sections of 16 WT and 6 HSD17B1TG mice at the age of 5.5 months were cut at 5 μm thickness and stained with hematoxylin and eosin. One uterine horn was cut into 4–5 pieces. The uterus piece second closest to the ovarian end was cut through and used for the analysis for each mouse. On average, 133 ± 9 and 220 ± 16 sections for WT and HSD17B1TG mice were analyzed, respectively. For the time point of 12 months, one slide per uterus, containing an average of 48 ± 5 sections for WT and 51 ± 6 for HSD17B1TG mice, was analyzed from the uterus piece second closest to the ovary. Endometrial glands partially or fully infiltrated into myometrium were considered as a sign for adenomyotic changes in all time points. All slides were scanned with Pannoramic 250 Flash II digital slide scanner (3DHISTECH, Budapest, Hungary). The gland infiltration depth was measured using the Caseviewer program (3DHISTECH, Budapest, Hungary).

### 4.3. Immunohistochemistry

For IHC, paraffin-embedded uteri were cut into 5 μm sections. The sections were deparaffinized and rehydrated in xylene and ethanol series. Antigen retrieval was performed in a pressure cooker (Retriever 1200) in Tris-EDTA (pH 9.0). The sections were blocked against non-specific binding in PBS–3% BSA–0.05% Tween and incubated with the primary antibody (monoclonal mouse anti-human smooth muscle actin clone 1A4, 71 μg/mL, Dako Agilent, Santa Clara, CA, USA) overnight. The endogenous peroxidase was then blocked by incubating the sections in 3% (*v*/*v*) H_2_O_2_ for 20 min. The primary antibody was detected by using an anti-mouse Dako Envision+ system (Dako Agilent, Santa Clara, CA, USA). Visualization was made with DAB+ substrate (Dako Agilent, Santa Clara, CA, USA). All sections were counterstained with Mayer’s hematoxylin and scanned with a Pannoramic 250 Flash II digital slide scanner (3DHISTECH, Budapest, Hungary).

### 4.4. RNA Isolation

Total RNA (WT *n* = 5, TG *n* = 5) was isolated using Trizol reagent (Thermo Fisher Scientific, Carlsbad, CA, USA), further purified with RNeasy columns (Qiagen, Germantown, MD, USA) and treated with DNase (RNase-free DNase Set, Qiagen; or DNase I, Invitrogen, Thermo Fisher Scientific, Carlsbad, CA, USA). The RNA concentrations were measured using Nanodrop ND-1000 spectrophotometer (Thermo Fisher Scientific, Carlsbad, CA, USA) and quality of RNA was verified using Bioanalyzer 2100 (Dako Agilent, Santa Clara, CA, USA).

### 4.5. RNA Sequencing and Data Analysis

The mRNA was sequenced with Illumina NovaSeq6000 at Novogene Cambridge Genomic Sequencing Centre (UK) with a standard protocol of paired-end 150 bp sequencing length. Sequencing depth was at least 20 million reads per sample and reads were mapped to mouse genome (Mus_musculus_Ensemble_94) using HISAT2 [54]. Differential expression analysis was conducted using DESeq2 [55]. Transcript abundance was quantified using FPKM (fragments per kilobase of transcript sequence per millions base pairs sequenced) [56]. For downstream differential expression analysis, we used FDR < 0.05, FC > 2, and genes for which mean FPKM was >1 in either HSD17B1TG or WT. The individual specific visualization of selected transcriptional changes was conducted using R 3.5 and FPKM. The sequencing results were submitted to GEO under the series accession number GSE199492.

Functional enrichment analysis (over-representation) was conducted in metascape.org with functional categories from GO Biological Processes (GO), Hallmark gene sets (H), canonical pathways (CP), KEGG (K), Reactome (R) and WikiPathways (W). Gene Set Enrichment Analysis (GSEA) (www.gsea-msigdb.org/gsea, accessed 19 January 2020) was conducted using HSD17B1TG versus WT transcriptional fold changes as the continuous ranked list and selected relevant gene list from the literature as custom datasets. These included differentially transcribed genes previously detected in the eutopic endometrium of human adenomyosis patients [21], estrogen treatment of mouse uterus [22] (FDR < 0.05, FC > 2) and siPGR-regulated genes [23].

### 4.6. RT-qPCR

For qPCR of *Klf4* and *Elf3*, 0.5 ug RNA (WT *n* = 11, TG *n* = 7) was transcribed to cDNA using the SensiFAST Kit (Bioline, Cincinnati, OH, US)). 0.1 ng/well of template cDNA was amplified using SYBR Green DyNamo HS Flash qPCR Kit (Thermo Fisher Scientific, Carlsbad, CA, USA). The primer sequencies were as follows: *Klf4* (forward 5′ -> 3′ TACCCTCCTTTCCTGCCAGA and reverse 5′ → 3′ CGGTAGTGCCTGGTCAGTTC, T_m_ = 60.0), *Elf3* (forward 5′ → 3′ CGGTGGAAGTGATGTGGACC and reverse 5′ → 3′ GTTCCAGGATCTCCCGTTTGT, T_m_ = 60.0).

### 4.7. Serum Hormone Analyses

The concentrations of serum progesterone, testosterone and E2 were analyzed by a validated gas chromatography–tandem mass spectrometry method [57] with the quantification limits of 74, 8 and 0.5 pg/mL, respectively.

### 4.8. HSD17B1 Inhibitor Treatment

The HSD17B1 inhibitor beta thiazole (compound no. 21 in Table 1 in Messinger et al., 2008 [24]) was delivered for the HSD17B1TG mice using custom pellets (Innovative Research of America) releasing 22 mg/kg/d of the inhibitor, and vehicle-treated mice were used as controls. The pellets were inoculated into mice anesthetized with 450 to 800 μL 2.5% tribromoethanol and 0.15 mg/kg buprenorphine, i.p. Postoperative analgesia was obtained by providing 0.1 mg/kg buprenorphine, s.c., daily for 3 postoperative days. The inhibitor dosing continued for 6 weeks starting at the age of 4 months after which the mice were sacrificed, and tissue samples were collected. The uterine horn was processed at the age of 5.5 months and 220 ± 16 and 215 ± 10 sections were analyzed for vehicle- and inhibitor-treated HSD17B1TG mice.

### 4.9. Statistical Analysis

For statistical analyses of the histological samples, GraphPad Prism software was used. Student’s t-test was applied for normally distributed data and the Mann–Whitney U-test for non-normally distributed datasets. Chi-square test was used to study the phenotypic frequencies.

## Data Availability

The RNA-seq data produced in this study are available in GEO under the series accession number GSE199492. Additional RNA-seq data used in the intersections were extracted from publications referenced in the methods. All the data and processed files are also available on request from the corresponding author.

## Supplementary Materials

The following supporting information can be downloaded at: https://www.mdpi.com/article/10.3390/ijms23094815/s1.
